# Pilot Study on the Effect of Patient Condition and Clinical Parameters on Hypoxia-Induced Factor Expression: *HIF1A*, *EPAS1* and *HIF3A* in Human Colostrum Cells

**DOI:** 10.3390/ijms252011042

**Published:** 2024-10-14

**Authors:** Julia Zarychta, Adrian Kowalczyk, Karolina Słowik, Dominika Przywara, Alicja Petniak, Adrianna Kondracka, Monika Wójtowicz-Marzec, Patrycja Słyk-Gulewska, Anna Kwaśniewska, Janusz Kocki, Paulina Gil-Kulik

**Affiliations:** 1 Student Scientific Society of Clinical Genetics, Medical University of Lublin, 20-080 Lublin, Poland; julia.zarychta99@gmail.com (J.Z.); adriankowalczyk31@gmail.com (A.K.); slowikarolina@o2.pl (K.S.); 2 Doctoral School, Medical University of Lublin, 20-093 Lublin, Poland; dprzywara17@gmail.com; 3 Department of Clinical Genetics, Medical University of Lublin, 20-080 Lublin, Poland; alicja.petniak@umlub.pl (A.P.); janusz.kocki@umlub.pl (J.K.); 4 Department of Obstetrics and Pathology of Pregnancy, Medical University of Lublin, 20-081 Lublin, Poland; adriannakondracka@wp.pl (A.K.); slyk.patrycja@gmail.com (P.S.-G.); anna.kwasniewska@umlub.pl (A.K.); 5 Chair and Department of Pediatric Nursing, Faculty of Health Sciences, Medical University of Lublin, 20-093 Lublin, Poland; monika.wojtowicz-marzec@umlub.pl

**Keywords:** hpoxia-inducible factor, HIF1A, EPAS1, HIF3A, human milk, colostrum, gene expression

## Abstract

Hypoxia-inducible factor 1 (HIF-1) may play a role in mammary gland development, milk production and secretion in mammals. Due to the limited number of scientific reports on the expression of HIF genes in colostrum cells, it was decided to examine the expression of *HIF1A*, *HIF3A* and *EPAS1* in the these cells, collected from 35 patients who voluntarily agreed to provide their biological material for research, were informed about the purpose of the study and signed a consent to participate in it. The expression of HIF genes was assessed using qPCR. Additionally, the influence of clinical parameters (method of delivery, occurrence of stillbirths in previous pregnancies, BMI level before pregnancy and at the moment of delivery, presence of hypertension during pregnancy, presence of *Escherichia coli* in vaginal culture, iron supplement and heparin intake during pregnancy) on the gene expression was assessed, revealing statistically significant correlations. The expression of *HIF1A* was 3.5-fold higher in the case of patients with the presence of *E. coli* in vaginal culture (*p* = 0.041) and 2.5 times higher (*p* = 0.031) in samples from women who used heparin during pregnancy. Approximately 1.7-fold higher expression of the *EPAS1* was observed in women who did not supplement iron during pregnancy (*p* = 0.046). To our knowledge, these are the first studies showing the relationship between HIF expression in cells from breast milk and the method of delivery and health condition of women giving birth. The assessment of HIF expression requires deeper examination in a larger study group, and the results of further studies will allow to determine whether HIF can become biomarkers in pregnancy pathology states.

## 1. Introduction

Hypoxia-inducible factor 1 (HIF-1) regulates the expression of genes involved in the adaptation of cells and tissues to low oxygen levels [1]. HIF-1 was discovered by Semenza et al. in 1992 as a transcription factor induced by hypoxia, which leads to a significant increase in the expression of the erythropoietin gene under hypoxia [2]. So far, three HIF-α isoforms have been discovered: the HIF-1α subunit, HIF-2α and HIF-3α, encoded by three separate genes (*HIF1A*, *EPAS1* and *HIF3A*) [3,4]. Despite the high homology between HIF-1α and HIF-2α, the expression of the second isoform is not as common as HIF-1α [5,6]. Additionally, it has been shown that the level of HIF-1α protein increases after exposure to oxygen deficiency in the acute phase of hypoxia and then gradually decreases. The level of HIF-2α increases and accumulates as a result of long-term hypoxia [7]. The functions and role of HIF-3α in hypoxia are not fully understood due to the presence of many isoforms of this protein. It is suggested that HIF-3α has a dual role: first, it inhibits the activity of HIF-1α and HIF-2α by competing for binding with HIF-1β subunits, and second, it regulates its own target genes such as *EPO*, *ANGPTLA4* and *GLUT1* [8,9].

In response to low oxygen levels, cells increase the expression and stability of HIF. HIF-1α and HIF-2α proteins dimerize with the HIF-1β isoform, which then binds to deoxyribonucleic acid sequences, promoting the expression of genes such as vascular endothelial growth factor (VEGF), placental growth factor and angiopoietins 1 and 2, which contribute to the growth of blood vessels [10]. Hypoxia is a typical feature of the early period of pregnancy because the physiological oxygen concentration in the uterine cavity during the first trimester is approximately 2–3% O_2_ [11]. The increased HIF-1 activity in this period is described as one of the most important regulators of placental vascularization and invasion [12]. Ietta et al. demonstrated dynamic HIF expression during placental development. HIF-1α is present throughout pregnancy its level peaks early (weeks 7–10) and then declines. HIF-2α expression is stable throughout pregnancy [13]. Recently, Colson et al. reported increased HIF-2α expression in the syncytium of pathological placentas in the second trimester of pregnancy, compared to placentas from normal pregnancies. They also suggested that excessive HIF-2α expression impairs placental function and thus contributes to preeclampsia and fetal growth restriction [14].

Seagroves et al. described the roles of HIF-1 in the mammary epithelium in the context of mammary gland development, milk production and secretion in mice. Mice lacking HIF-1α inhibited epithelial cell differentiation, ultimately leading to decreased milk synthesis and altered milk composition [15]. Cells of the mammary gland epithelium (MEC) take up glucose, which is the main substrate for milk production, through glucose transporters (GLUT) [16]. Shao et al. demonstrated that in mice, the expression level of GLUT1 is regulated through a mechanism dependent on Hif-1α [17]. Hif-1α signaling is linked to hypoxia resulting from the high metabolic activity in the mammary gland during the early stages of pregnancy and throughout the lactation period [18]. Hif-1 is responsible for the upregulation of angiogenic factors such as VEGF, which, under hypoxic conditions, induces angiogenesis and increases oxygen availability in the endothelium [19]. Research on hypoxia in the mammary gland during lactation is less common than that related to breast cancer. Both during lactation and in malignant growth, the metabolism of the breast undergoes changes [20]. However, the role of hypoxia and the transcriptional response of HIF-1α in promoting tumor progression and metastasis is well established [21,22,23], while the effects of hypoxia on lactation in the mammary gland have yet to be defined.

Due to the probable significant involvement of *HIF* genes in milk production, as well as the influence of these genes on the functioning of the placenta and fetal growth mentioned in the literature, it was decided to evaluate the expression of these genes in human colostrum cells due to the high content of bioactive ingredients in mother’s milk in the initial period of lactation [24].

The aim of our study was to test hypotheses regarding the gene expression of *HIF* in the cellular fraction of breast milk and the relationship between this expression and the health status of mothers. The study assessed the expression level of the *HIF1A*, *EPAS1* and *HIF3A* genes depending on clinical parameters such as the health of the pregnant woman, the method of delivery, the occurrence of stillbirths in previous pregnancies, the occurrence of hypertension during pregnancy, Body Mass Index (BMI) values before and during pregnancy, the intake of iron preparations during pregnancy, taking heparin during pregnancy and the presence of *E. coli* in vaginal culture, because it is the most common cause of bloodstream infections during pregnancy [25].

To the best of our knowledge, this is the first study assessing the level of HIF-1α expression in the cellular fraction of breast milk and providing an analysis of gene expression depending on the health status of pregnant women.

## 2. Results

### 2.1. Study Group

A total of 35 patients took part in the study on the third day after delivery, aged 18–44 (mean age 30.9 ± 5), hospitalized at the Department of Obstetrics and Pathology of Pregnancy of the Independent Public Clinical Hospital No. 1 in Lublin. On the third day after delivery, in the morning (up to two hours after a meal), 5 mL of milk was collected from each of the examined patients into a sterile container.

Only patients without addictions, whose children received 10 points on the Apgar scale after birth and whose adaptation was progressing correctly, were qualified for the study group (study inclusion criteria) [26]. The exclusion criteria from the study were: addictions in the mother, especially smoking and improper adaptation of the newborn, including breathing disorders. A total of 16 patients (46%) had a vaginal delivery, while 19 patients (54%) had a cesarean section. The women gave birth to 22 boys and 13 girls. 60% of the study group (N = 21) were healthy patients, not suffering from chronic diseases (control group). The remaining 14 patients had concomitant diseases such as hypertension (N = 6), diabetes (N = 5) and hypothyroidism (N = 8). Five patients had diabetes and hypothyroidism at the same time. Four (11%) of 35 patients had a history of stillbirth. The characteristics of the patients qualified for the study are presented in Supplementary Tables S1 and S2. Each patient underwent a vaginal smear before delivery, and a microbiological test was ordered; the test protocol and the vaginal culture result are presented in Supplementary Table S2.

### 2.2. Gene Expression Analysis

The analyses performed did not show a significant relationship between the expression of the *HIF1A*, *EPAS1* and *HIF3A* genes in milk cells with: maternal age, the presence of diabetes and hypothyroidism in the mother, week of gestation, laboratory test results of the mother and child, the child’s gender and the child’s birth weight.

The analyses that showed significant relationships are described in the following subsections. Supplementary Table S3 shows the analyses for which a significant relationship between the tested clinical factor and at least one tested gene was shown. The table shows the size of the compared groups, the average expression level, the standard deviation and the result of the statistical test.

### 2.3. Influence of the Method of Delivery

**Figure 1:**
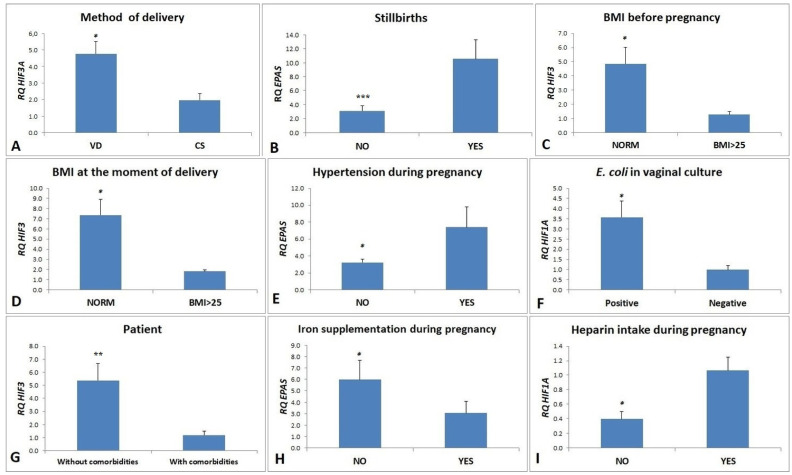
(**A**) Average expression of the *HIF3* gene (RQ ± SE) in the cellular fraction of breast milk depending on the method of delivery, (**B**) Average expression of the *EPAS1* gene (RQ ± SE) in the cellular fraction of breast milk depending on the occurrence of stillbirths in previous pregnancies, (**C**) Average expression of the *HIF3A* gene (RQ ± SE) in the cellular fraction of breast milk depending on the BMI before pregnancy, (**D**) Average expression of the *HIF3A* gene (RQ ± SE) in the cellular fraction of breast milk depending on the BMI at the moment of delivery, (**E**) Average expression of the *EPAS1* gene (RQ ± SE) in the cellular fraction of breast milk depending on the occurrence of hypertension during the pregnancy, (**F**) Average expression of the *HIF1A* gene (RQ ± SE) in the cellular fraction of breast milk depending on the presence of *Escherichia coli* in vaginal culture, (**G**) Average expression of the *HIF3* gene (RQ ± SE) in the cellular fraction of breast milk depending on the presence of comorbidities, (**H**) Average expression of the *EPAS1* gene (RQ ± SE) in the cellular fraction of breast milk depending on the iron supplementation by the patient, (**I**) Average expression of the *HIF1A* gene (RQ ± SE) in the cellular fraction of breast milk depending on the heparin treatment, * *p* < 0.05, ** *p* < 0.01, *** *p* ≤ 0.001 as determined by a Mann–Whitney U test.

It was observed that the expression of the *HIF3A* gene was nearly 2.5 times higher (Figure 1A) in milk collected from patients giving birth by vaginal delivery than in women giving birth by cesarean section (*p* = 0.043).

### 2.4. Influence of the Occurrence of Stillbirths

Expression of the EPAS1 gene was more than three times higher in the material collected from women with a history of stillbirths in previous pregnancies than in the cellular fraction of milk collected from women without a history of stillbirths (*p* = 0.001) (Figure 1B).

### 2.5. Influence of the BMI Level before Pregnancy

**Figure 2:**
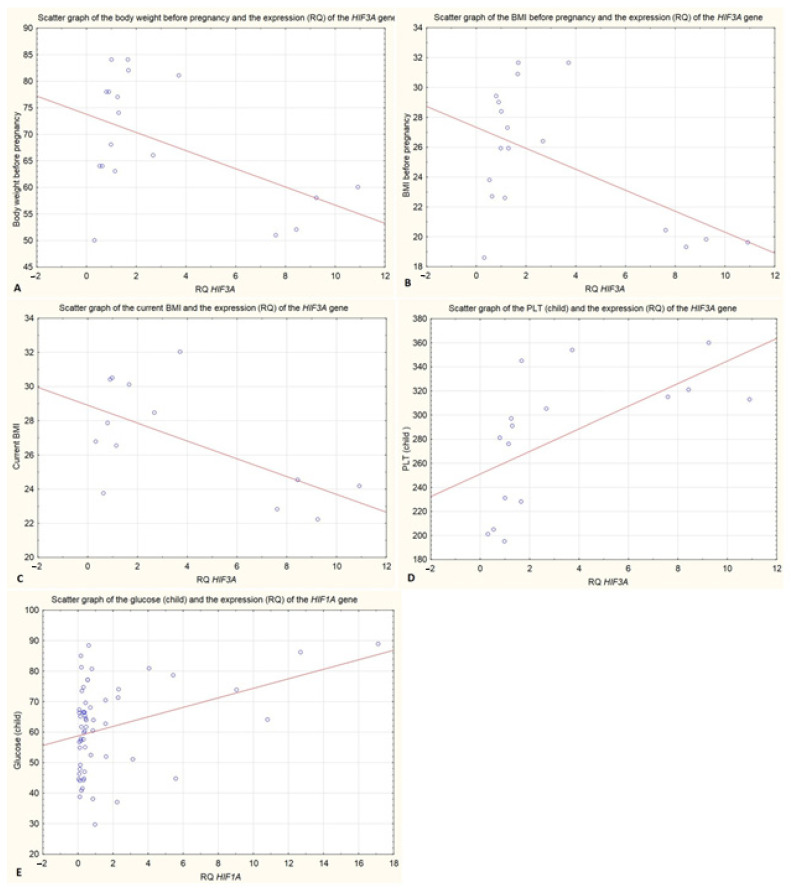
(**A**) Scatter graph of the body weight before pregnancy and the expression (RQ) of the *HIF3A* gene in the cellular fraction of breast milk (r = −0.509 *p* < 0.05); (**B**) Scatter graph of the BMI before pregnancy and the expression (RQ) of the *HIF3A* gene in the cellular fraction of breast milk (r = −0.509 *p* < 0.05); (**C**) Scatter graph of the current BMI and the expression (RQ) of the *HIF3A* gene in the cellular fraction of breast milk (r = −0.509 *p* < 0.05); (**D**) Scatter graph of the PLT (child) and the expression (RQ) of the *HIF3A* gene in the cellular fraction of breast milk (r = −0.509 *p* < 0.05); (**E**) Scatter graph of the glucose (child) and the expression (RQ) of the *HIF1A* gene in the cellular fraction of breast milk (r = −0.509 *p* < 0.05) Spearman’s Rank Correlation.

In the case of an analysis of *HIF3A* gene expression in the cellular fraction of breast milk, an almost 4-fold lower expression of this gene was observed in patients with BMI above 25 kg/m^2^ before pregnancy (*p* = 0.032) (Figure 1C). Spearman’s correlation test showed that the expression of the *HIF3A* gene is statistically significantly negatively correlated with the BMI value before pregnancy (r = −0.540, *p* < 0.005) (Table 1), (Figure 2).

### 2.6. Influence of the BMI Level at the Moment of Delivery

In the case of *HIF3A* gene expression, a statistically significant difference was observed (*p* = 0.033), and the expression of this gene was over five times higher in the cellular fraction of breast milk collected from patients with normal BMI (Figure 1D). There was also a statistically significant negative correlation between *HIF3A* expression and BMI (r = −0.615, *p* < 0.005) (Table 1) (Figure 2).

### 2.7. Influence of the Presence of Hypertension during Pregnancy

In the case of *EPAS1* gene expression, it was found to be more than two-fold higher in the material collected from patients with hypertension during pregnancy (*p* = 0.031) (Figure 1E).

### 2.8. Influence of the Presence of E. coli in Vaginal Culture

In the case of *HIF1A* gene expression, it was more than 3.5-fold higher in the material collected from patients with the presence of *E. coli* in vaginal culture (*p* = 0.041) (Figure 1F).

### 2.9. Influence of the Iron Supplement Intake during Pregnancy

Approximately 1.7-fold higher expression of the *EPAS1* gene was observed in women who did not supplement iron during pregnancy (*p* = 0.046) (Figure 1H).

### 2.10. Influence of the Heparin Intake during Pregnancy

*HIF1A* gene expression was statistically significantly more than 2.5 times higher (*p* = 0.031) in samples from women who used heparin during pregnancy (Figure 1I).

## 3. Discussion

### 3.1. The Method of Delivery Affects HIF3A Gene Expression

In the case of elective cesarean sections (performed before the onset of labor), the level of O_2_ saturation in the capillary blood of the myometrium is higher than in the case of women who underwent an emergency cesarean section due to a lack of progress in natural labor [27]. During vaginal delivery, due to the high contractility of the uterine myometrium leading to episodes of vessel lumen closure within it, periods of endometrial hypoxia occur, which contribute to the increase in HIF-1α level [28,29]. Wen et al. showed that under hypoxic conditions, HIF-1α contributes to maintaining the proper contractility of the uterine myometrium [29]. The role of HIF-1α in the metabolic adaptation of endometrial cells to the processing of chemical compounds in order to generate the energy needed for contraction in hypoxic conditions is also indicated. HIF-1α, by binding to the serpin family E member 1 (*SERPINE1*) promoter, activates the expression of a gene whose protein product, through interaction with ATP synthase peripheral stalk subunit F6 (ATP5PF), allows maintaining ATP production in the oxidative phosphorylation pathway despite transient periods of hypoxia [30].

To our best knowledge, no relationship between *HIF3A* gene expression and method of delivery has been reported in the literature so far. However, the results of our study may suggest that HIF-3α, similar to HIF-1α, plays a role in maintaining the proper contractility of the uterine myometrium during labor in hypoxic conditions, but confirmation of this finding requires further research.

It has also been reported that in the menstrual phase, the level of HIF-1α increases in the endometrium, which plays a role in its repair [31,32]. To our best knowledge, the role of HIF-1α in endometrial regeneration after childbirth has not yet been described. However, regardless of the method of delivery, endometrium damage occurs during delivery [33]. In line with our expectations, the level of HIF-1α was similar in patients giving birth vaginally and by cesarean section (no statistically significant difference was observed between these groups), which may suggest that HIF-1α also mediates the regeneration of damaged endometrium after giving birth, but the confirmation of this finding requires further research.

It should be noted that our studies were performed in the milk of women on the third day after delivery, which sheds new light on the influence of the route of delivery on the quality of mother’s milk. It is likely that the milk of mothers giving birth vaginally will have a different effect on the newborn in the context of *HIF* expression than the milk of mothers giving birth by cesarean section. This is a new and unexplored topic that requires further explanation.

### 3.2. The Occurrence of Stillbirths Affects EPAS1 Gene Expression

The development of the fetus in the mother’s uterus is a complex process. The placenta plays a key role in keeping the fetus alive by enabling the exchange of nutrients and gases between the mother and her child. Therefore, the disruption of the structure or function of the placenta may result in impaired fetal growth, miscarriage or stillbirth [34,35]. HIF-1α has been shown to play an important role in the early stage of pregnancy, enabling the proper development of the embryo and fetus. HIF-1α has been reported to be involved in endometrial receptivity by regulating glycolysis [31,36].

Yu et al. showed that the expression of *HIF1A* in the peri-implantation endometrium is lower in women with recurrent implantation failure (RIF) compared to women without a history of RIF [37]. Another extremely important aspect is the development of maternal-fetal tolerance, thanks to which the mother’s immune system does not attack the cells of the fetus and placenta. In the early stage of pregnancy, the placenta is in an oxygen-poor environment, which results in the increased expression of *HIF1A* [38]. Köstlin-Gille et al. demonstrated that increased *HIF1A* expression contributes to the accumulation of myeloid-derived suppressor cells (MDSC) in the uterus and their activation. MDSCs, by exerting an immunosuppressive effect on other cells of the immune system, contribute to the development of immunological tolerance to fetal tissues [38].

The results of our research indicate the involvement of HIF-2α in maintaining pregnancy at its later stages. To our best knowledge, there are no reports in the literature about the role of HIF-2α in the late stage of pregnancy. So far, only the role of HIF-2α in the embryo implantation process has been confirmed [37].

Matsumoto et al. indicated that HIF-2α, by promoting the expression of genes encoding membrane-type metalloproteinase 2, lysyl oxidase, VEGF and adrenomedulin, is involved in the detachment of the luminal epithelium and thus supports the invasion of the blastocyst trophectoderm into the endometrium and activation of an embryonic survival signal [37].

The discovery of the role of HIF-2α at the late stage of pregnancy requires further research, which seems particularly important because molecular mechanisms involved in the physiological process of maintaining pregnancy have not yet been fully elucidated. Elucidating these mechanisms could contribute to better perinatal care and the development of more effective methods of preventing stillbirths, especially since the stillbirth rate in highly developed countries has not improved significantly recently (stillbirth rates in North America—3.3 in 2000 and 2.7 in 2021; stillbirth rates in Western Europe—3.9 in 2000 and 2.6 in 2021) [39,40].

Taking into account the increased expression of *EPAS1* in women with a history of stillbirth, the measurement of HIF-2α during pregnancy could have a diagnostic role and allow the selection of a group of women at risk of stillbirth, which may result in providing specialized care to such patients. It should be noted, however, that the material used in this study came from four women staying in the Clinic of Obstetrics and Pathology of Pregnancy who had a history of stillbirth; this is only 11% of the study group. This led to a significant disparity between the compared groups. Therefore, to validate the results, it is necessary to conduct the analysis on a larger sample of patients. Additionally, the expression of *EPAS1* was examined in the milk of women after delivery, which is a significant limitation of the study. The topic requires deeper examination and assessment of *HIF* expression, for example in the blood serum of pregnant women at various stages of pregnancy.

### 3.3. The BMI Level Affects HIF3A Gene Expression

BMI over 25 kg/m^2^ is defined by the World Health Organization as pre-obesity, while BMI over 30 is defined as obesity class I [41]. Obesity has been shown to promote the development of a pro-inflammatory environment in the placenta [42,43]. Children born to obese mothers are more likely to suffer from pneumonia and are more likely to suffer from macrosomia [44].

Obesity also negatively affects the mother’s health. Hypertrophy of adipocytes, as well as insufficient vascularization in relation to the needs, promotes the occurrence of local hypoxia in adipose tissue, which in turn leads to an increase in the level of HIF-1α, which mediates the development of insulin resistance [45]. Insulin is one of the factors that upregulates *HIF3A* expression [46], so it was expected that patients with higher BMI would show greater *HIF3A* gene expression. However, the results of our study proved the opposite; the level of *HIF3A* gene expression was lower. This may be because hyperglycemia and high fatty acid concentrations destabilize HIF-1α [47]. A similar mechanism may occur with HIF-3α. This is supported by other studies showing reduced expression of *HIF3A* in women with gestational diabetes [48].

In addition, a correlation has been observed between *HIF3A* promoter methylation and gestational diabetes [48]. This is consistent with our study, wherein it was shown that *HIF3A* negatively correlates with women’s BMI. These results align with Dick et al. research, where they indicated that higher BMI correlates with heightened methylation at the *HIF3A* locus in both blood cells and adipose tissue. The increase in *HIF3A* gene methylation was inversely proportional to its expression [49].

It has been reported that HIF-3α, by regulating the expression of *LIPE, PLIN1* and *PNPLA2* genes in adipocytes, may contribute to regulating the lipolysis process. A detailed explanation of the mechanisms by which HIF-3α is involved in the regulation of metabolic processes may be important for the development of effective strategies for the pharmacological prevention or treatment of obesity [50].

Moreover, the expression of *HIF3A* in cells from breast milk may have long-term effects on the health of the child. Mansell at all. showed a relationship between *HIF3A* promoter methylation in umbilical cord blood and the blood pressure of a child at 4 years of age [51]. Cells from breast milk have the ability to penetrate from the gastrointestinal tract into the circulation of the child and then become incorporated into the structure of various organs. Thus, it can be speculated that a mother’s BMI may affect the blood pressure of her offspring even in late childhood, but this hypothesis needs confirmation in further studies.

Breast milk has a wide ability to adapt to the baby’s needs; it cannot be ruled out that the changed expression of *HIF* genes is a kind of compensation for the newborn’s current needs, but this hypothesis requires further research.

### 3.4. The Occurrence of Hypertension during Pregnancy Affects EPAS1 Gene Expression

HIF-2α levels increase due to prolonged hypoxia [7]. Thus, it is understandable why *EPAS1* gene expression is higher in women suffering from a chronic disease such as hypertension. Moreover, the occurrence of increased *EPAS1* expression may influence the pathogenesis of hypertension.

There are many reports in the literature linking the expression of *EPAS1* with the development of pulmonary hypertension in the event of alveolar hypoxia, but to our best knowledge, no mechanism has been demonstrated so far in which *EPAS1* could contribute to the development of arterial hypertension [52,53]. However, it is worth noting that HIF-2α mediates the increase in the expression of endothelin 1 (*EDN1*) [53]. Chan et al. in their work generated smooth muscle cells with a heterozygous *EPAS1* gain-of-function mutation. Increased *EPAS1* expression contributed to increased *EDN1* expression, which then caused stiffening of smooth muscle cells [54]. One of the components influencing the regulation of blood pressure is peripheral vascular resistance. The increase in vessel wall contractility caused by abnormal EDN1 expression contributes to increased peripheral resistance and thus plays a role in the pathogenesis of hypertension [55].

### 3.5. The Presence of E. coli in Vaginal Culture Influences HIF1A Gene Expression

The occurrence of an infection in the body can trigger the onset of hypoxia, which in turn increases HIF activity [56]. Studies on mice lacking HIF-1α in immune cells assessed the impact of infection and inflammation on the immune system. It has been shown that HIF-1α plays a key role in host defense against pathogens such as *Helicobacter pylori*, *Mycobacterium tuberculosis* and *Escherichia coli* [57,58,59]. This is supported by our study, which showed an increase in *HIF1A* expression in milk obtained from women who were found to be vaginally colonized with *E. coli* bacteria.

The correlation between increased levels of HIF-1α and ongoing infection has been repeatedly reported in the literature. Mimouna et al. showed that adherent-invasive *E. coli* (AIEC) strains promote an increase in the cellular level of HIF-1α, which in turn, through its involvement in autophagy induction, contributes to the removal of AIEC [60,61]. Similar results were achieved by Cane et al., who showed that the infection of human Afa/Dr cells with diffusely adhering *E. coli* C1845 strain induces *HIF1A* expression in intestinal epithelial cells [62].

Increased *HIF1A* expression in infected tissues is caused by the exposure of cells to bacterial lipopolysaccharides (LPS) and low pH [63,64]. Yang et al. presented evidence in their work that *E. coli* LPS in a normoxic environment induces calcium signaling, which regulates the expression of the *HIF1A* gene via the signal transducer and activator of transcription 3 (STAT3) pathway. Additionally, calcium present intracellularly through the Endothelin 1—Endothelin receptor type A (ET-1-EdnrA) pathway increases the stability of HIF-1α [65]. It is worth noting that in the case of infection, not only LPS stimulates the expression of genes encoding HIF-1α, but also local metabolic acidosis resulting from the infection [63]. The disturbance of the citric acid cycle caused by too low pH promotes the accumulation of 2-Hydroxyglutarate, which stabilizes HIF-1α by competing with α-Ketoglutarate for the binding site with PHD [63,66]. Our results indicating the increased expression of the *HIF1A* gene in the case of vaginal colonization by *E. coli* are therefore consistent with the results obtained by other researchers.

Modulating the level of HIF-1α in infected cells may be one of the potential therapeutic solutions to alleviate recurrent infections. This is indicated by the works of Bandarra et al. and Lin et al., who proved, respectively, that HIF-1α, by regulating nuclear factor kappa-light-chain-enhancer of activated B cells (NF-κB) activity, contributes to maintaining the immune balance by counteracting excessive pro-inflammatory reactions in response to infection and may also contribute to improving the effectiveness of the innate antibacterial response [59,65].

Thus, it can be speculated that breast milk from mothers who have vaginal colonization by *E. coli* bacteria may possess stronger anti-inflammatory properties compared to breast milk from mothers without the infection.

### 3.6. EPAS1 Gene Expression Is Affected by Iron Supplement Intake during Pregnancy

The post-transcriptional regulation of the activity of the HIF-α subunit is mediated by the PHD enzyme, which is dependent on the Fe^2+^ concentration [65]. HIF-1 consists of two subunits—an unstable α subunit and a stable β subunit. The regulation of alpha chain stability is mediated by prolyl hydroxylase (PHD), which belongs to the superfamily of Fe^2+^ and α-ketoglutarate-dependent oxygenases [5]. Under aerobic conditions, PHD leads the alpha subunit to ubiquitination and degradation in the proteasome. In a situation of hypoxia, the inhibition of this process causes HIF-1α to stabilize, dimerize with the HIF-1β subunit, translocate to the cell nucleus and bind to hypoxia response elements in the promoter regions of various genes involved in erythropoiesis, angiogenesis, cell proliferation and survival, as well as glucose and iron metabolism [3,4,67]. Thanks to the constant transcription and translation of HIF-1α and a lack of the need for de novo protein synthesis, a rapid cell response to hypoxia is possible [67]. There are many reports in the literature confirming that iron deficiency, by limiting the activity of the PHD enzyme, stabilizes HIF-1α by increasing its level in the cell [68,69,70,71]. A similar relationship was described in the case of HIF-2α by Kong et al., who showed in their work that the use of deferoxamine, an iron chelator, stabilizes HIF-2α [5].

In the study, it was decided to check whether iron supplementation during pregnancy will affect the expression of genes encoding isoforms of the HIF-α subunit or whether the regulation of HIF-α subunit activity by iron concentration takes place only at the post-transcriptional level. The results of our research indicate that the expression of the *EPAS1* gene, encoding HIF-2α, is regulated by iron at the transcriptional level. Lower expression of the *EPAS1* gene was found in the cellular fraction of milk from patients who took iron during pregnancy vs. patients who did not supplement iron. Our results are consistent with those of Oshima et al., who, in a study on mice, showed that iron supplementation reduces the expression of the gene encoding HIF-2α in the kidneys of rodents [72].

### 3.7. HIF1A Gene Expression Is Affected by Heparin Intake during Pregnancy

During pregnancy, the risk of thromboembolic events, including deep vein thrombosis and pulmonary embolism, increases. International guidelines on the care of pregnant women support the use of heparin as part of antithrombotic prophylaxis after analyzing the risk factors present in the pregnant woman [73].

There are no reports in the literature regarding the correlation between heparin intake and the expression of genes encoding HIF-α subunit isoforms. It was speculated that the increased *HIF1A* gene expression in patients taking heparin during pregnancy may be related to their predisposition to thromboembolic events. A thrombus appearing in the vessel wall limits blood flow and therefore may lead to hypoxia, which in turn is a factor that increases HIF-1α activity [74]. Evans et al. showed that *HIF1A* was expressed in the mouse thrombosed inferior vena cava, and increasing the concentration of HIF1-α in the vein wall through the use of a PHD domain inhibitor promoted thrombus resolution [75,76]. However, currently, the role of HIF-1α in the resolution of venous thrombus is not clear [74].

The expression of the gene encoding *VEGF* is regulated by HIF-1α, and researchers indicate that there may be a causal relationship between increased VEGF levels and the occurrence of venous thromboembolism [77,78]. Therefore, conducting further research on the role of HIF-1α in thromboembolic events seems important due to the potential therapeutic use of modulating HIF-1α activity in the treatment of thromboembolic events.

The aim of our work was to assess the level of HIF-1α expression in the cellular fraction of breast milk and provide an analysis of gene expression depending on the health status of pregnant women. To our knowledge, this has not been the subject of research so far. Therefore, it was decided to conduct preliminary analyses to determine *HIF* expression in breast milk depending on clinical factors and whether *HIF* expression can be used with a view to predicting obstetric diseases/failures in pregnant women or possible complications occurring in mothers after childbirth. The limitation of our study is that it was conducted on a small number of patients; the *p* values obtained in the study for some comparisons are close to 0.05, so the research should be extended to include a larger and more homogeneous study group; second the single collection of milk samples 3 days after delivery. However, studies report that long-term conditions of hypoxia (which may occur in anemia requiring iron supplementation), hypertension or obesity may have a long-term impact on the expression of individual genes and cause the accumulation of their expression products [79,80,81]. The research of Seagroves et al. shows that HIF-1α plays a key role in the functioning of the mammary epithelium and lactation, and that is why it was decided to assess whether various pathological conditions occurring during pregnancy will change the expression of *HIF* genes in the milk of mothers after delivery [15]. Due to the promising results of our preliminary study, in the next stages of the experiment, it is planned to closely monitor the expression of *HIF* in the blood of pregnant women with particular diseases during pregnancy, compared to the control group, which will make it possible to assess whether *HIF* can be used to diagnose or predict the occurrence of diseases in pregnant women. The results of our preliminary study allowed us to select diseases in which the probability of using *HIF* as a marker seems to be higher.

## 4. Materials and Methods

### 4.1. Ethics Approval and Sample Collection

The research was conducted with the consent of the Bioethics Committee at the Medical University of Lublin (KE-0254/88/04/2022). Each patient gave informed, written consent to participate in the study. The material used for the study was human milk, collected from 35 patients.

### 4.2. Sample Size

The sample size for the study was selected based on clinical parameters, which include the method of delivery, the occurrence of stillbirths in previous pregnancies, the BMI level before and at the time of delivery, hypertension during pregnancy, the presence of *E. coli* in vaginal culture, iron supplementation and taking heparin during pregnancy. Additionally, a sample size calculator was used to calculate the sample size for the study, with the margin of error being 5%. Taking into account the above-mentioned parameters, the sample size for this preliminary study was selected. Due to the nature of the study (preliminary report), it was decided to examine the correlations with 35 patients.

### 4.3. Isolation of Total Cellular Ribonucleic Acid (RNA) from Breast Milk Samples

Milk samples were collected by the patient after previous instructions in the morning, 2 h after a meal, into a sterile container. Immediately after collection, fresh milk samples were transported to the Department of Clinical Genetics of the Medical University of Lublin for further analyses. The collected milk in the amount of 5 mL was centrifuged for 20 min at a speed of 805× *g* at a temperature of 15 °C (5810R Eppendorf centrifuge). After centrifugation, the supernatant and the fatty phase were collected, and the cellular fraction was washed by centrifugation twice with buffered saline (PBS, Biomed, Lublin, Poland). The cell pellet was stored at −80 °C until RNA isolation, but for no longer than 2 weeks.

Total cellular RNA was extracted from the total milk cell population (obtained from centrifugation of 5 mL of whole milk) using the mirVanaTM miRNA isolation kit (Invitrogen by Thermo Fisher Scientific, Vilnius, Lithuania). The isolation procedure followed the reagent manufacturer’s instructions. After isolation, the RNA was stored at −80 °C until the next stage of the study.

The NanoDrop 2000c device (Thermo Scientific, Waltham, MA, USA) and the electrophoretic technique (Agilent Bioanalyzer 2100 Agilent Technologies, Santa Clara, CA, USA) were used to perform qualitative and quantitative analysis of the isolated RNA.

### 4.4. Complementary DNA (cDNA) Synthesis in Reverse Transcription Reaction

The reverse transcription (RT) reaction was then performed using the commercially available High-Capacity cDNA Transcription Kits with RNase Inhibitor (Applied Biosystems, Vilnius, Lithuania). The reaction was performed in a volume of 20 μL according to the manufacturer’s protocol. For the RT reaction, the following samples were taken 1 µg of isolated RNA. The reverse transcription reaction was performed with a Verit Thermal Cycler (Applied Biosystems, Foster City, CA, USA), consisting of a mixture of reagents (1 µL RNase 40 U/µL; 1 µL reverse transcriptase 50 U/µL; 2 µL 10× RT buffer; 3.2 µL ultrapure water; 0.8 µL 10× dNTP (100 mM); 2 µL 10× RT Random Primer; 1 μg RNA dissolved in 10 μL ultrapure water). The reaction mixture was incubated at 25 °C for 10 min, sequentially at 37 °C for 2 h and then at a temperature of 95 °C for 5 min. The obtained cDNA was used for qPCR.

### 4.5. Real-Time Polymerase Chain Reaction (PCR)

The real-time PCR reaction was performed using the StepOnePlus System (Applied Biosystems, Waltham, MA, USA), which consisted of: 1 µL of cDNA after the reverse transcription reaction; ultrapure water free from RNase and DNase; Gene Expression Master Mix (Applied Biosystems, Vilnius, Lithuania) and a probe specific for the tested gene. The following molecular probes were used:

1. TaqMan HIF1A: Hs00153153_m1 NM_001243084.1
2. TaqMan HIF3A: Hs00541709_m1; NM_022462.4;
3. TaqMan EPAS1: Hs01026149_m1; NM_001430.4;
4. GAPDH (Hs99999905_m1; NM_001289746.1) (Applied Biosystems by Thermo fisher Scientific, Pleasanton, CA, USA) was used as an endogenous control.

qPCR reactions were performed using the Step One Plus system (Applied Biosystems, Waltham, MA, USA). using reverse transcription cDNA as a template, in 96-well plates with a capacity of 0.1 mL (Applied Biosystems, Waltham, MA, USA), in a volume of 10 µL/well, consisting of the reaction mixture (0.5 µL gene-specific primers and probes (Applied Biosystems by Thermo fisher Scientific, Pleasanton, CA, USA); 5 µL TaqMan Gene Expression Master Mix (Applied Biosystems, Vilnius, Lithuania) and 4.5 µL cDNA synthesized by reverse transcription with ultrapure RNAse- and DNase-free water). After an initial 10-min denaturation at 95 °C, the reaction was carried out for 40 cycles according to the following scheme: 15 s at 95 °C, then 60 s at 60 °C. The expression of the studied genes was analyzed using Expression Suite v.1.0.3 software (Life Technologies, Waltham, MA, USA). Three technical repetitions were performed on each tested sample. Three endogenous controls were used during the study (*GAPDH, B2M* and *18S*); the most stable control was GAPDH used to assess the expression of the studied genes. A negative control was used. The detailed course of the validated reaction and the procedures used to assess gene expression have been described in detail in our previous works [82,83,84].

### 4.6. Evaluation of the Expression Level of the Studied Genes

The relative amount of the product after the reaction (or gene expression in the tested sample relative to the control sample, which means the fold change in the expression of the tested gene in the tested sample relative to the control sample) was calculated using Livak’s method [85]. RQ= 2^−∆∆Ct^. The results of GAPDH-normalized gene expression: *HIF1A*, *EPAS1* and *HIF3A* in the tested material were analyzed using Expression Suite Software v1.0.3 (Life technologies, Waltham, MA, USA). RQ (Relative quantification).

### 4.7. Statistical Analysis

Statistical analysis was performed using Statistica v.13 software. Due to the fact that the distribution of the studied samples significantly differed from the normal distribution (Shapiro–Wilk W test), the analyses were performed using nonparametric tests. The Mann–Whitney U test was used to assess differences between the research groups, and the Spearman test was used to assess correlation. Correction for multiple comparisons was not performed. The adopted level of statistical significance is *p* < 0.05.

### 4.8. Evaluation of Clinical Data

Doctors of the Department of Obstetrics and Pathology of Pregnancy of the Independent Public Clinical Hospital No. 1 in Lublin assessed clinical data of the examined patients and their newborn children, which are presented in Supplementary Tables S1 and S2.

## 5. Conclusions

In summary, our study provides new information on the effect of maternal health conditions on the expression of *HIF1A*, *EPAS1* and *HIF3A* genes. It was shown that *HIF3A* gene expression is higher in the cells from breast milk of mothers who gave birth vaginally, compared to women who gave birth by cesarean section. In addition, *EPAS1* expression is higher in women who have a history of stillbirths in their births. It was also observed that there is a positive correlation between *EPAS1* expression and the occurrence of stillbirths. In addition, there was lower expression of the *HIF3A* gene for mothers whose BMI was higher than 25 kg/m^2^ both during and before pregnancy. *HIF3A* was also negatively correlated with BMI.

Moreover, the higher expression of *EPAS1* in cells from the milk of mothers with hypertension during pregnancy and those who did not supplement iron during pregnancy was shown. In addition, the higher expression of *HIF1A* in the case of material obtained from women with vaginal colonization by *E. coli* bacteria, as well as those who took heparin during pregnancy, was observed.

To our knowledge, these are the first studies showing the relationship between *HIF* expression in cells from breast milk and the method of delivery and health condition of women giving birth. Our results shed new light on the properties of breast milk, but the assessment of HIF expression requires deeper examination in a larger study group, and the results of further studies will allow to determine whether HIF can become biomarkers in pregnancy pathology states.

It is worth emphasizing that the altered expression of HIF family genes in milk may not only be a potential marker of the mother’s health but also have important clinical implications for the newborn. Higher expression of HIF family genes supports cells in survival in a low-oxygen environment, has the ability to stimulate the expression of genes responsible for erythropoiesis, angiogenesis, metabolic adaptation, or anti-inflammatory properties. This suggests that the change in HIF expression in milk, depending on the patient’s and delivery parameters, will also translate into the properties of milk and its effect on the newborn. This indicates a new direction in research on mother’s milk.

## Figures and Tables

**Table 1:** Analysis of the relationship between analyzed genes and clinical data, * *p* < 0.05 Spearman’s Rank Correlation.

Parameter	*RQ EPAS1*	*RQ HIF1A*	*RQ HIF3A*
*RQ EPAS1*	1.000	0.681 *	0.483 *
*RQHIF1A*	0.681 *	1.000	−0.090
*RQ HIF3A*	0.483 *	−0.090	1.000
Apgar score	0.002	0.086	−0.382
PLT (mother)	−0.095	0.132	0.077
HGB (mother)	−0.198	−0.038	0.190
WBC (mother)	−0.067	−0.010	0.204
Glucose (mother)	−0.069	0.264	0.111
CRP (mother)	−0.178	−0.180	–
Body weight of the newborn	−0.033	−0.088	−0.356
PLT (child)	0.134	−0.168	0.608 *
HGB (child)	−0.182	−0.014	0.100
WBC (child)	−0.076	−0.192	0.144
Total bilirubin (child)	−0.099	0.007	−0.328
Glucose (child)	0.019	0.356 *	0.231
CRP (child)	−0.064	−0.054	−0.054
Pregnancy—order	−0.045	0.009	0.186
Number of miscarriages	0.053	0.100	0.201
Number of children alive	−0.014	−0.015	0.050
Week of gestation	−0.076	−0.066	0.110
Age	−0.220	0.059	0.166
Height	−0.125	0.122	0.230
Body weight before pregnancy	−0.029	−0.127	−0.509 *
BMI before pregnancy	0.028	−0.180	−0.540 *
Current body weight	0.198	0.029	−0.500
Difference in body weight	−0.214	0.098	0.235
Current BMI	0.095	−0.096	−0.615 *

PLT—platelet count; HGB—hemoglobin; WBC—white blood cell count; CRP—C-reactive protein; BMI—Body Mass Index.

## Data Availability

The data used to support the findings of this study are included in the article.

## Supplementary Materials

The following supporting information can be downloaded at: https://www.mdpi.com/article/10.3390/ijms252011042/s1, References [86,87,88] are cited in the Supplementary Materials.
