# Supplementary material for: Pilot Study on the Effect of Patient Condition and Clinical Parameters on Hypoxia-Induced Factor Expression: HIF1A, EPAS1 and HIF3A in Human Colostrum Cells
Source: Int J Mol Sci. 2024 Oct 14;25(20):11042. doi: 10.3390/ijms252011042 (PMC11507067; doi:10.3390/ijms252011042)
Supplement: Supplementary file 1 [file ijms-25-11042-s001.zip › ijms-3198616-supplementary.pdf]

Table S1. Characteristics of the study group in terms of patient's age, week of pregnancy in which delivery occurred and clinical parameters of mother and child.

Parameter	N*	Mean	Minimum	Maximum	SD
Pregnancy - order	35	2.122	1.000	7.000	1.3028
Number of miscarriages	35	0.378	0.000	3.000	0.7347
Occurence of stillbirths	35	0.068	0.000	1.000	0.2527
Week of gestation	35	38.623	31.000	41.000	1.7010
Age [years]	35	30.865	18.000	44.000	5.0918
Height [cm]	35	166.108	150.000	186.000	6.3947
Body weight before pregnancy	35	64.010	46.000	98.500	10.0903
BMI before pregnancy	35	23.164	17.374	32.000	3.3906
Current body weight [kg]	24	74.286	57.000	94.000	8.6751
Current BMI	24	27.230	21.551	32.526	3.1766
CRP (mother) [mg/l]	15	10.185	0.600	101.000	22.2198
Glucose (mother) [mg/dl]	35	78.812	9.740	108.000	26.9672
WBC (mother) [x 109/l.]	35	10.944	5.660	18.400	2.6487
HGB (mother) [g/dl]	35	12.327	10.010	14.600	0.9557
PLT (mother) [x 109/l.]	35	224.753	114.000	377.000	58.8146
CRP (child) [mg/l]	16	5.448	0.600	41.100	8.8802
Glucose (child) [mg/dl]	35	61.379	29.600	88.800	14.1889
Total bilirubin (child)	16	8.324	0.090	19.070	3.1349
WBC (child) [x 109/l.]	16	14.909	6.900	28.710	5.7312
HGB (child) [g/dl]	16	17.327	12.400	21.100	1.8876
PLT (child) [x 109/l.]	16	278.939	158.000	394.000	60.8819
Body weight (child) [g]	35	3452.500	1150.000	4350.000	541.6875

* *For some parameters. data for all 35 patients were not obtained.* BMI – Body Mass Index; CRP – C-reactive protein; WBC – white blood cell count; HGB - hemoglobin; PLT – platelet count; SD – standard deviation.

Table S2. Numbers in the study group depending on clinical parameters such as: method of delivery, occurrence of stillbirths in previous pregnancies, the sex of the newborn, vaginal culture result, comorbidities during pregnancy, medicines taken during pregnancy.

**Characteristics Of The Study Group**		
**Parameter**	**N**	**%**
**Method Of Delivery**		
VD	16	46
CS	19	54
**Stillbirths**		
NOS	31	89
YES	4	11
**Sex Of The Newborn**		
M	13	37
F	22	63
**Vaginal Culture**		
Lactobacillus		
YES	7	20
NO	28	80
E. coli		
YES	7	20

NO	28	80
Staphylococcus spp.		
YES	4	11
NO	31	89
Streptococcus agalactiae		
YES	6	17
NO	29	83
Candida albicans		
YES	4	11
NO	31	89
**Comorbidities During Pregnancy**		
**Diabetes**		
YES	5	14
NO	30	86
**Hypertension**		
YES	6	17
NO	29	83
**Hypothyroidism**		
YES	8	23
NO	27	77
**Inflammation Of The Urinary Tract**		
YES	6	17
NO	29	83
**Genital Tract Infection**		
YES	10	29
NO	25	71
**Respiratory Tract Infection**		
YES	5	14
NO	30	86
**Medicines Taken During Pregnancy**		
**Heparin**		
YES	5	14
NO	30	86
**Acard**		
YES	4	11
NO	31	8
**Magnesium**		
YES	24	69
NO	11	31
**Lutein**		
YES	14	40
NO	21	60
**Medicines For Hypertension**		
YES	6	17
NO	29	83
**Iron**		
YES	24	69
NO	11	31

VD – vaginal delivery; CS – caesarean section; M – male; F – female.

Vaginal Culture

As part of the standard diagnostic procedure at the Department of Obstetrics and Pathology of Pregnancy of the Independent Public Clinical Hospital No. 1 in Lublin, each patient admitted for delivery at the time of admission to the ward had a vaginal swab performed. Specimens were taken with the use of commercial sterile cotton swabs by the medical staff. During the procedure there was no contact with external genital organs, perineal and anal areas. All swabs were placed on a transport base and directly transported to the hospital's laboratory. Samples were placed on anaerobic Blood agar and MacConkey agar plates, which were then incubated at 37°C. The growth of bacterial colonies was assessed after 24 and 48 hours. Then, bacterial isolates collected from the colonies were selected for further identification in accordance with the Polish guidelines [1-3].

[1] Safarpoor Dehkordi F, Tavakoli-Far B, Jafariaskari S, Momtaz H, Esmaeilzadeh S, Ranjbar R, Rabiei M. Uropathogenic Escherichia coli in the high vaginal swab samples of fertile and infertile women: virulence factors, O-serogroups, and phenotyping and genotyping characterization of antibiotic resistance. New Microbes New Infect. 2020 Nov 24;38:100824. doi: 10.1016/j.nmni.2020.100824. PMID: 33364031; PMCID: PMC7750135.
[2] Ghartey JP, Carpenter C, Gialanella P, Rising C, McAndrew TC, Mhatre M, Tugetman J, Einstein MH, Chazotte C, Herold BC. Association of bactericidal activity of genital tract secretions with Escherichia coli colonization in pregnancy. Am J Obstet Gynecol. 2012 Oct;207(4):297.e1-8. doi: 10.1016/j.ajog.2012.07.025. Epub 2012 Jul 26. PMID: 22867687; PMCID: PMC3462306.
[3] Tsekouras N, Meletis E, Kostoulas P, Labronikou G, Athanasakopoulou Z, Christodoulopoulos G, Billinis C, Papatsiros VG. Detection of Enterotoxigenic Escherichia coli and Clostridia in the Aetiology of Neonatal Piglet Diarrhoea: Important Factors for Their Prevention. Life (Basel). 2023 Apr 27;13(5):1092. doi: 10.3390/life13051092. PMID: 37240738; PMCID: PMC10223568.

Table S3. Average expression of the examined genes (RQ±SD) in the cellular fraction of breast milk depending on: the method of delivery, occurrence of stillbirths in previous pregnancies, BMI before pregnancy, BMI at the moment of delivery, hypertension during pregnancy, *Escherichia coli* in vaginal culture, patient with comorbidities, iron supplementation, heparin treatment * $p<0.05$ ** $p<0.01$ *** $p\leq0.001$ Mann Whitney U test.

**Method Of Delivery**							
**Gene**	**VD**			**CS**			**p**
	**N**	**Mean**	**SD**	**N**	**Mean**	**SD**	
RQ EPAS1	16	4.044	4.808	19	3.491	4.901	0.662
RQ HIF1A		1.332	2.601		1.796	3.497	0.55
RQ HIF3A		4.765	3.367		1.965	2.322	**0.043***
**Occurrence Of Stillbirths In Previous Pregnancies**							
**Gene**	**No**			**Yes**			**p**
	**N**	**Mean**	**SD**	**N**	**Mean**	**SD**	
RQ EPAS1	31	3.122	2.89	4	10.537	9.955	**0.001*****
RQ HIF1A		1.56	3.172		1.892	2.425	0.82
RQ HIF3A		3.054	3.446		4.206	3.587	0.456
**BMI Before Pregnancy**							
**Gene**	**Norm**			**BMI>25**			**p**
	**N**	**Mean**	**SD**	**N**	**Mean**	**SD**	

Gene	N	Mean	SD	N	Mean	SD	p
RQ EPAS1		3.978	5.411		3.669	3.505	0.858
RQ HIF1A	29	1.749	3.382	6	1.558	2.592	0.861
RQ HIF3A		4.864	3.582		1.28	0.647	**0.032***
**BMI At The Moment Of Delivery**							
**Gene**	**Norm**			**BMI>25**			**p**
	**N**	**Mean**	**SD**	**N**	**Mean**	**SD**	
RQ EPAS1		3.263	2.384		3.803	3.346	0.682
RQ HIF1A	18	2.089	3.721	17	1.67	2.748	0.774
RQ HIF3A		7.374	3.955		1.828	1.313	**0.033***
**Hypertension During Pregnancy**							
**Gene**	**No**			**Yes**			**p**
	**N**	**Mean**	**SD**	**N**	**Mean**	**SD**	
RQ EPAS1		3.256	3.722		7.406	6.551	**0.031***
RQ HIF1A	21	1.593	3.241	6	1.526	2.04	0.955
RQ HIF3A		3.295	3.586		1.121	0.808	0.417
***Escherichia coli* In Vaginal Culture**							
**Gen**	**No**			**Yes**			**p**
	**N**	**Mean**	**SD**	**N**	**Mean**	**SD**	
RQ EPAS1		2.664	2.363		1.33	0.57	0.148
RQ HIF1A	28	0.999	2.157	7	3.578	6.526	**0.041***
RQ HIF3A		3.249	3.618		1.301	0	0.609
**Patient**							
**Gene**	**Healthy**			**With Comorbidities**			**p**
	**N**	**Mean**	**SD**	**N**	**Mean**	**SD**	
RQ EPAS1		2.131	1.782		3.602	6.496	0.337
RQ HIF1A	21	0.36	0.235	14	0.622	0.915	0.222
RQ HIF3A		5.376	4.139		1.196	0.692	**0.006****
**Iron Supplementation**							
**Gene**	**No**			**Yes**			**p**
	**N**	**Mean**	**SD**	**N**	**Mean**	**SD**	
RQ EPAS1		5.376	7.577		3.038	2.835	**0.046***
RQ HIF1A	11	1.285	1.781	24	1.706	3.515	0.622
RQ HIF3A		2.968	3.418		3.123	3.652	0.928
**Heparin Treatment**							
**Gene**	**No**			**Yes**			**p**
	**N**	**Mean**	**SD**	**N**	**Mean**	**SD**	
RQ EPAS1		2.672	4.595		3.859	5.392	0.601
RQ HIF1A	30	0.396	0.549	5	1.065	1.042	**0.031***
RQ HIF3A		3.271	3.712		1.966	1.529	0.565

VD – vaginal delivery; CS – caesarean section
